# Molecular Generation for Desired Transcriptome Changes With Adversarial Autoencoders

**DOI:** 10.3389/fphar.2020.00269

**Published:** 2020-04-17

**Authors:** Rim Shayakhmetov, Maksim Kuznetsov, Alexander Zhebrak, Artur Kadurin, Sergey Nikolenko, Alexander Aliper, Daniil Polykovskiy

**Affiliations:** ^1^Insilico Medicine, Hong Kong, Hong Kong; ^2^Neuromation OU, Tallinn, Estonia

**Keywords:** deep learning, generative models, adversarial autoencoders, conditional generation, representation learning, drug discovery, gene expression

## Abstract

Gene expression profiles are useful for assessing the efficacy and side effects of drugs. In this paper, we propose a new generative model that infers drug molecules that could induce a desired change in gene expression. Our model—the Bidirectional Adversarial Autoencoder—explicitly separates cellular processes captured in gene expression changes into two feature sets: those *related* and *unrelated* to the drug incubation. The model uses *related* features to produce a drug hypothesis. We have validated our model on the LINCS L1000 dataset by generating molecular structures in the SMILES format for the desired transcriptional response. In the experiments, we have shown that the proposed model can generate novel molecular structures that could induce a given gene expression change or predict a gene expression difference after incubation of a given molecular structure. The code of the model is available at https://github.com/insilicomedicine/BiAAE.

## Introduction

Following the recent advances in machine learning, deep generative models found many applications in biomedicine, including drug discovery, biomarker development, and drug repurposing (Mamoshina et al., 2016; Zhavoronkov, 2018). A promising approach to drug discovery is conditional generation, where a machine learning model learns a distribution *p*(*x* | *y*) of molecular structures *x* with given property *y*. Such models can generate molecules with a given synthetic accessibility, binding energy, or even activity against a given protein target (Kadurin et al., 2016; Polykovskiy et al., 2018a).

In this paper, we studied how conditional models scale to a more complex biological property; specifically, we have studied how drug incubation influences gene expression profiles. Using the LINCS L1000 (Duan et al., 2014) dataset, we build a joint model *p*(*x*, *y*) on molecular structures *x* and induced gene expression changes *y*.

In many conditional generation tasks, *x* completely defines *y*. For example, molecular structure completely defines its synthetic accessibility score. For our task, however, some transcriptome changes are unrelated to the drug effect on cells, and we cannot infer them only from an incubated drug.

We propose a new model—the Bidirectional Adversarial Autoencoder—that learns a joint distribution *p*(*x*, *y*) of objects and conditions. The model decomposes objects and their properties into three feature parts: shared features *s* common to both *x* and *y*; exclusive features *z_x_* relevant only to *x* and not *y*; and exclusive features *z_y_* relevant only to *y* and not *x*: *p*(*x*, *y*) = *p*(*s*, *z_x_*, *z_y_*). For the transcriptomes and drugs, shared features *s* may contain pharmacophore properties, target protein information, binding energy, and inhibition level; exclusive features *z_x_* may describe the remaining structural information; and *z_y_* may represent unrelated cellular processes. As features *s* are common to both *x* and *y*, the model can extract them from both *x* and *y*.

The paper is organized into sections: *Related Work* surveys related work; *Models* presents the proposed Bidirectional Adversarial Autoencoder; *Experimental Evaluation* compares and validates the models on two datasets: the toy Noisy MNIST dataset of hand-written digits and LINCS L1000 dataset of small molecules with corresponding gene expression changes; and *Conclusion* concludes the paper.

## Related Work

Conditional generative models generate objects *x* from a conditional distribution *p*(*x* | *y*), with *y* usually being limited to class labels. The Adversarial Autoencoder (AAE) (Makhzani et al., 2015) consists of an autoencoder with a discriminator on the latent representation *z* that tries to make the latent space distribution indistinguishable from a prior distribution *p*(*z*); its conditional extension—Supervised AAE (Makhzani et al., 2015)—works well for simple conditions but can violate the conditions in other cases (Polykovskiy et al., 2018b). Conditional Generative Adversarial Networks (CGAN) (Mirza and Osindero, 2014) supplied the condition as an auxiliary input to both generator and discriminator. Perarnau et al. (2016) inverted CGANs, allowing us to edit images by changing the labels *y*. In FusedGAN (Bodla et al., 2018), a GAN generated a generic “structure prior” with no supervision, and a CGAN generated an object *x* from condition *y* and the latent representation learned by the unconditional GAN. Other papers explored applications of Conditional AAE models to the task of image modification (Antipov et al., 2017; Lample et al., 2017; Zhang et al., 2017).

CausalGAN (Kocaoglu et al., 2018) allowed components of the condition to have a dependency structure in the form of a causal model making conditions more complex. The Bayesian counterpart of AAE, the Variational Autoencoder (VAE) (Kingma and Welling, 2013), also had a conditional version (Sohn et al., 2015a), where conditions improved structured output prediction. CycleGAN (Zhu et al., 2017) examined a related task of object-to-object translation.

Multimodal learning models (Ngiam et al., 2011) and multi-view representation models (Wang et al., 2016a) explored translations between different modalities, such as image to text. Wang et al. (2016b) presented a VAE-based generative multi-view model. Our Bidirectional Adversarial Autoencoder provided explicit decoupling of latent representations and brought the multi-view approach into the AAE framework, where the basic Supervised AAE-like models (Makhzani et al., 2015) did not yield correct representations for sampling (Polykovskiy et al., 2018b).

Information decoupling ideas have been previously applied in other contexts: Yang et al. (2015) disentangled identity and pose factors of a 3D object; adversarial architecture from Mathieu et al. (2016) decoupled different factors in latent representations to transfer attributes between objects; Creswell et al. (2017) used VAE architecture with separate encoders for class label *y* and latent representation *z*, forcing *z* to exclude information about *y*; InfoVAE (Zhao et al., 2017) maximized mutual information between input and latent features; and Li et al. (2019) proposed a VAE modification that explicitly learns a “disentangled” representation *s* to predict the class label and a “non-interpretable” representation *z* that contains the rest of the information used for decoding.

InfoGAN (Chen X. et al., 2016) maximized mutual information between a subset of latent factors and the generator distribution. FusedGAN (Bodla et al., 2018) generated objects from two components, where only one component contains all object-relevant information. Hu et al. (2018) explicitly disentangles different factors in the latent representation and maps a part of the latent code to a particular external information.

### Conditional Generation for Biomedicine

Machine learning has numerous applications in biomedicine and drug discovery (Gawehn et al., 2016; Mamoshina et al., 2016; Ching et al., 2018). Deep neural networks demonstrated positive results in various tasks, such as prediction of biological age (Putin et al., 2016; Mamoshina et al., 2018a; Mamoshina et al., 2019), prediction of targets and side effects Aliper et al., 2017; Mamoshina et al., 2018b; West et al., 2018), and applications in medicinal chemistry (Lusci et al., 2013; Ma et al., 2015).

Alongside large-scale studies that measure cellular processes, deep learning applications explore transcriptomics (Aliper et al., 2016b; Chen Y. et al., 2016); these works study cellular processes and their change following molecular perturbations. Deep learning has also been applied to pathway analysis (Ozerov et al., 2016), the prediction of protein functions (Liu, 2017), the discovery of RNA binding proteins (Zheng et al., 2017), the discovery of binding patterns of transcription factors (Qin and Feng, 2017), medical diagnostics based on omics data (Chaudhary et al., 2017), and the analysis of DNA and RNA sequences (Budach and Marsico, 2018).

In drug discovery, apart from predicting pharmacological properties and learning useful representations of small molecules (Duvenaud et al., 2015; Aliper et al., 2016a; Kuzminykh et al., 2018), deep learning is being widely applied to the generation of molecules (Sanchez and Aspuru-Guzik, 2018). Multiple authors have published models that generate new molecules that are similar to the training data or molecules with predefined properties (Kadurin et al., 2017a; Kadurin et al., 2017b; Segler et al., 2017 Gómez-Bombarelli et al., 2018). AI-generated molecules have also been tested *in vitro* (Polykovskiy et al., 2018b). Reinforcement learning and generative models further enabled the generation of complex non-differentiable objectives, such as novelty (Guimaraes et al., 2017; Putin et al., 2018a; Putin et al., 2018b). Generative models aim to eliminate the bottleneck of traditional drug development pipelines by providing promising new lead molecules for a specific target and automating the initial proposal of lead molecules with desired properties. Recently, Zhavoronkov et al. (2019) developed a model GENTRL to discover potent inhibitors of discoidin domain receptor 1 (DDR1) in 21 days.

## Models

In this section, we introduce Unidirectional and a Bidirectional Adversarial Autoencoders and discuss their applications to conditional modeling. While we have focused on an example of molecular generation for transcriptome changes, in general, our model is not limited to these data types and can be used for generation in other domains.

### Supervised Adversarial Autoencoder

**Figure 1:**
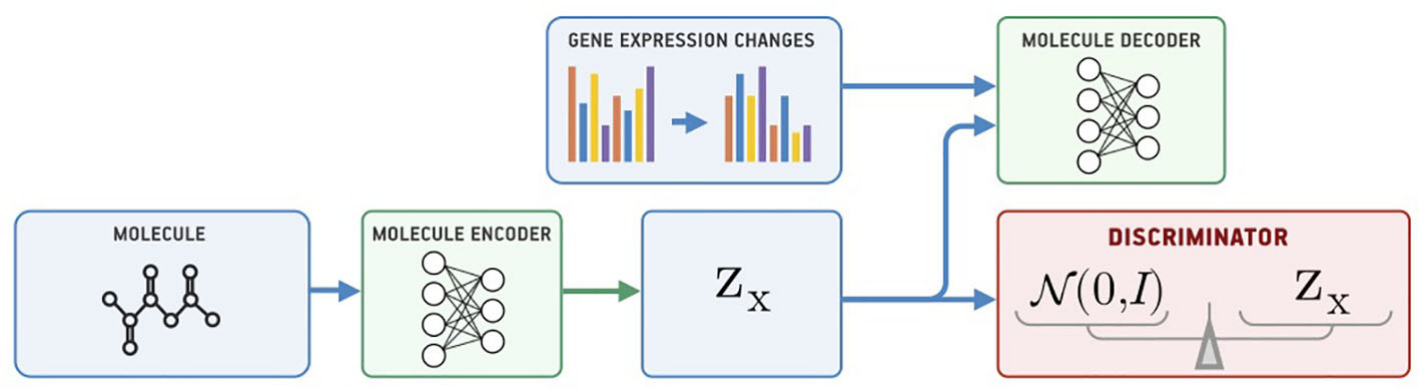
The Supervised Adversarial Autoencoder model (SAAE).

Our model for conditional generation is based on a Supervised Adversarial Autoencoder (Supervised AAE, SAAE) (Makhzani et al., 2015) shown in **Figure 1**. The Supervised AAE learns three neural networks—an encoder *E_x_*, a generator (decoder) *G_x_*, and a discriminator *D*. The encoder maps a molecule *x* onto a latent representation *z* = *E_x_*(*x*), and a generator reconstructs the molecule back from *z* and gene expression changes *y*: *G_x_*(*z, y*). We trained a discriminator *D* to distinguish latent codes from samples of the prior distribution *p*(*z*) and modified the encoder to make the discriminator believe that encoder’s outputs are samples from the prior distribution:

(1) $$\underset{E_{x},G_{x}}{\min}\;\underset{D}{\max}\;\lambda_{1}E_{x,y∼p_{\text{d}}\left(x,y\right)}l_{\text{rec}}^{x}\left(x,G_{x}\left(E_{x}\left(x\right),y\right)\right)+E_{z∼p\left(z\right)}\log\;D\left(z\right)+E_{x∼p_{\text{d}}\left(x\right)}\log\;(1-D\left(E_{x}\left(x\right)\right)),$$

where $l_{\text{rec}}^{x}$ is a similarity measure between the original and reconstructed molecule, and *p*_d_(*x*, *y*) is the data distribution. Hyperparameter λ_1_ balances reconstruction and adversarial losses. We trained the model by alternately maximizing the loss in Equation 1 with respect to the parameters of *D* and minimizing it with respect to the parameters of *E_x_* and *G_x_* (Goodfellow et al., 2014).

**Figure 2:**
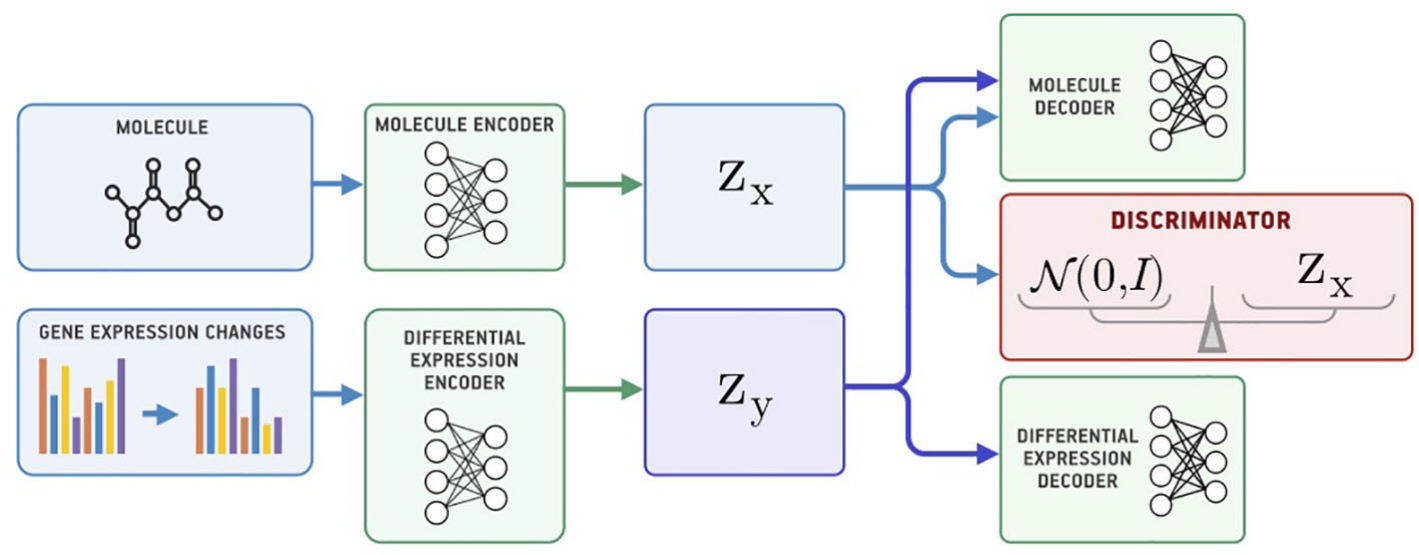
The Latent Supervised Adversarial Autoencoder model (Latent SAAE).

Besides passing gene expression changes *y* directly to the generator, we could also train an autoencoder (*E_y_*, *G_y_*) on *y* and pass its latent codes to the molecular decoder *G_x_* (**Figure 2**). We call this model a Latent Supervised Adversarial Autoencoder (Latent SAAE). Its optimization problem is:

(2) $$\underset{E_{x},E_{y},G_{x},G_{y}}{\min}\underset{D}{\max}\;\lambda_{1}E_{x,y∼p_{\text{d}}\left(x,y\right)}l_{\text{rec}}^{x}\left(x,G_{x}\left(E_{x}\left(x\right),E_{y}\left(y\right)\right)\right)+\lambda_{2}E_{y∼p_{\text{d}}\left(y\right)}l_{\text{rec}}^{y}\left(y,G_{y}\left(E_{x}\left(y\right)\right)\right)+E_{z∼p\left(z\right)}\log\;D\left(z\right)+E_{x∼p_{\text{d}}\left(x\right)}\log\;(1-D\left(E_{x}\left(x\right)\right)).$$

Hyperparameters λ_1_ and λ_2_ balance object and condition reconstruction losses as well as the adversarial loss.

### Bidirectional Adversarial Autoencoder

**Figure 3:**
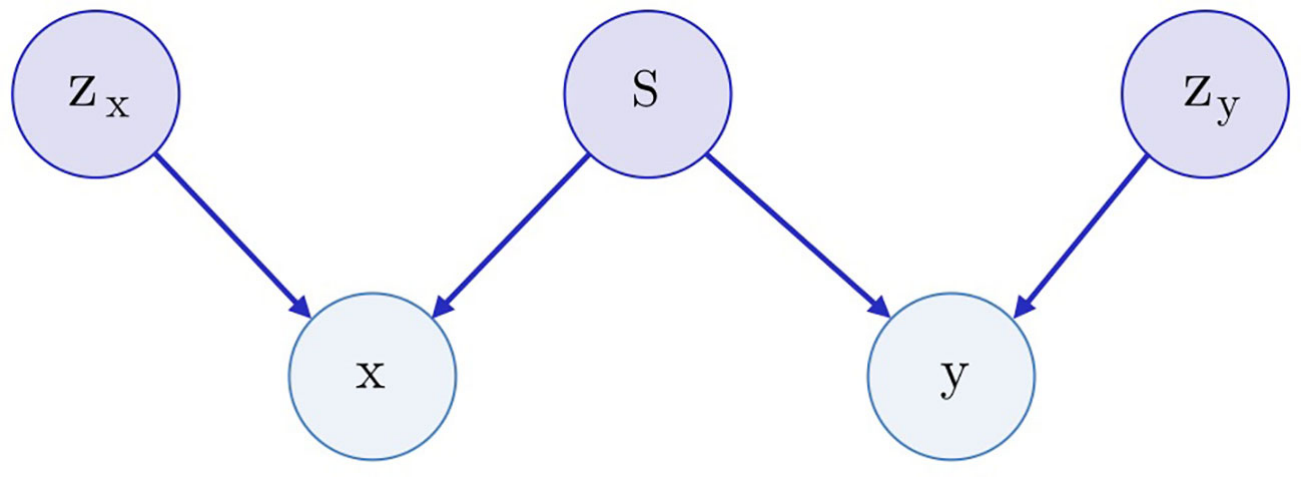
The underlying graphical model of the data: molecules *x*, gene expression changes *y*, three latent variables correspond to the exclusive (*z*_*x*_, *z*_*y*_) and shared (*s*) features between *x* and *y*.

Both SAAE and Latent SAAE models learn conditional distribution *p*(*x* | *y*) of molecules for specific transcriptome changes. In this paper, we learned a joint distribution *p*(*x*, *y*) instead. Our model is symmetric in that it can generate both *x* for a given *y* and *y* for a given *x*. We assume that the data are generated with a graphical model shown in **Figure 3**. Latent variables *z_x_* and *z_y_* are exclusive parts that represent features specific only to molecules or transcriptome changes. Latent variable *s* represents a shared part that describes features significant for both molecules and expression changes. To produce a new data point, we sampled exclusive (*z_x_*, *z_y_*) and shared (*s*) parts independently and used generative distributions *G_x_* (*x* | *s*, *z_x_*) and *G_y_* (*y* | *s*, *z_y_*) to produce *x* and *y*.

To train a model, we used inference networks that predict values of *s*, *z_x_*, and *z_y_*: *E_x_*(*z_x_* **|** *x*), *E_y_*(*z_y_* **|** *y*), and *E*(*s* **|** *x, y*) = *E_x_*(*s* **|** *x*) = *E_y_*(*s* **|** *y*). Note that we used two separate networks for inference of *s* from one of *x* and *y* to perform conditional sampling (when only one of *x* or *y* is known). For example, to sample *p*(*x* | *y*), we would do the following steps:

(3) $$s∼E_{y}(s|y),\;z_{x}∼p\left(z_{x}\right),\;x∼G_{x}\left(s,z_{x}\right).$$

For the molecule, *s* may describe its pharmacophore—binding points that are recognized by macromolecules. For the gene expression, *s* may describe affected proteins. Note that we can infer pharmacophore from a list of affected genes and vice versa. The exclusive part *z_x_* of a molecule describes the remaining structural parts besides the pharmacophore points. The exclusive part *z_y_* of a transcriptome describes cellular processes that influence the expression but are not caused by the drug.

**Figure 4:**
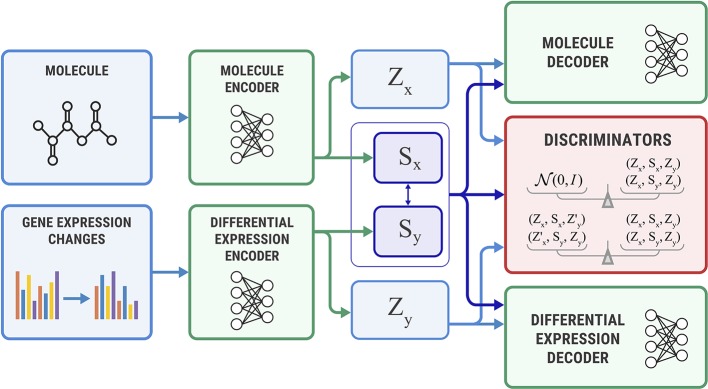
The Bidirectional Adversarial Autoencoders model. The discriminators ensure that three latent code components are independent and indistinguishable from the prior distribution.

**Figure 4** shows the proposed Bidirectional AAE architecture. We used two deterministic encoders *E_x_* and *E_y_* that infer latent codes from molecules and transcriptomes:

(4) $$\left(z_{x},s_{x}\right)=E_{x}\left(x\right),\;\left(z_{y},s_{y}\right)=E_{y}\left(y\right).$$

Two deterministic decoders (generators) *G_x_* and *G_y_* reconstruct molecules *x* and gene expression changes *y* back from the latent codes:

(5) $$x=G_{x}\left(z_{x},s_{x}\right),\;y=G_{y}\left(z_{y},s_{y}\right)$$

The objective function consists of three parts, each capturing restrictions from the graphical model—the structure of the shared representation, reconstruction quality, and independence of shared and exclusive representations.

**Shared loss** ensures that shared representations extracted from the molecule *s_x_* and gene expression *s_y_* are close to each other, as suggested by the graphical model:

(6) minEx,Ey ℒshared=Ex,y∼Pd(x,y) ‖sx−sy‖22.

**Reconstruction loss** ensures that decoders reconstruct molecules and gene expressions back from the latent codes produced by the encoders. We also use a cross-reconstruction loss, where molecular decoder *E_x_* uses shared part *s_y_* from a gene expression encoder *E_y_* for reconstruction and vice versa:

(7) $$\underset{E_{x},E_{y},G_{x}}{\min}{ℒ}_{\text{rec}}^{x}=E_{x∼p_{\text{d}}\left(x\right)}l_{\text{rec}}^{x}\left(x,G_{x}\left(z_{x},s_{x}\right)\right)+E_{x,y∼p_{\text{d}}\left(x,y\right)}l_{\text{rec}}^{x}\left(x,G_{x}\left(z_{x},s_{y}\right)\right)$$

(8) $$\underset{E_{x},E_{y},G_{y}}{\min}{ℒ}_{\text{rec}}^{y}=E_{y∼p_{\text{d}}\left(y\right)}l_{\text{rec}}^{y}\left(y,G_{x}\left(z_{y},s_{y}\right)\right)+E_{x,y∼p_{\text{d}}\left(x,y\right)}l_{\text{rec}}^{y}\left(y,G_{y}\left(z_{x},s_{y}\right)\right)$$

where $l_{\text{rec}}^{x}$ and $l_{\text{rec}}^{y}$ are some distance measures in the molecules and gene expression space.

**Discriminator loss** is an objective that encourages distributions *p*(*s*), *p*(*z_x_*), and *p*(*z_y_*) to be independent, which means that shared and exclusive parts must learn different features. This restriction comes from a graphical model. It also encourages *p*(*s*), *p*(*z_x_*), and *p*(*z_y_*) to be standard Gaussian distributions *N*(0, *I*) to perform a sampling scheme from Equation 3. We optimized the discriminator in an adversarial manner (Goodfellow et al., 2014) similar to SAAE:

(9) $$\underset{E_{x},E_{y},G_{x},G_{y}}{\min}\underset{D}{\max}{ℒ}_{\text{adv}}=E_{s^{\prime },{z^{\prime }}_{x},{z^{\prime }}_{y}∼p\left(s\right)p\left(z_{x}\right)p\left(z_{y}\right)}\log\;D\left({z^{\prime }}_{x},s^{\prime },{z^{\prime }}_{y}\right)+\frac{1}{2}E_{x,y∼p_{\text{d}}\left(x,y\right)}\log\left(\;1-D(z_{x},s_{x},z_{y})\right)+\frac{1}{2}E_{x,y∼p_{\text{d}}\left(x,y\right)}\log\;\left(1-D(z_{x},s_{y},z_{y})\right)$$

Note that since the target distribution for adversarial training is factorized, we expected that the trained model would learn independence of *s*, *z_x_*, and *z_y_*.

**Additional discriminator losses** We also added additional discrimination objective to explicitly encourage independence of *z_x_* from (*s_y_*, *z_y_*) and *z_y_* from (*s_x_*, *z_x_*):

(10) $$\underset{E_{x},E_{y},G_{x},G_{y}}{\min}\underset{D}{\max}{ℒ}_{\text{info}}=E_{x,y∼p_{\text{d}}\left(x,y\right)}E_{y^{\prime }∼p_{\text{d}}\left(y\right)}\left[\log\;D\left(z_{x},s_{x},z_{y}\right)+\log\left(1-D\;(z_{x},s_{x},{z^{\prime }}_{y})\right)\right]+E_{x,y∼p_{\text{d}}\left(x,y\right)}E_{x^{\prime }∼p_{\text{d}}\left(x\right)}\left[\log\;D\left(z_{x},s_{y},z_{y}\right)+\log\;\left(1-D\;({z^{\prime }}_{x},s_{y},z_{y})\right)\right],$$

where ${z^{\prime }}_{x}$ is an exclusive latent code of *x*′, and ${z^{\prime }}_{y}$ is an exclusive latent code of *y*′. In practice, we obtain *z_x_*′ and *z_y_*′ by shuffling *z_x_* and *z_y_* in each batch.

Combining these objectives, the final optimization problem becomes a minimax problem that can be solved by alternating gradient descent with respect to encoder and decoder parameters, and gradient ascent with respect to the discriminator parameters:

(11) $$\underset{E_{x},E_{y},G_{x},G_{y}}{\min}\underset{D}{\max}\lambda_{1}{ℒ}_{\text{shared}}+\lambda_{2}{ℒ}_{\text{rec}}^{x}+\lambda_{3}{ℒ}_{\text{rec}}^{y}+{ℒ}_{\text{adv}}+{ℒ}_{\text{info}}.$$

The hyperparameters λ_1_, λ_2_, and λ_3_ balance different objectives. In general, we optimize lambdas based on the performance of BiAAE on the holdout set in terms of the target metrics, such as estimated negative conditional log-likelihood. In practice, we found that optimal values of lambdas yielded the gradients of loss components on a similar scale.

### Unidirectional Adversarial Autoencoder

**Figure 5:**
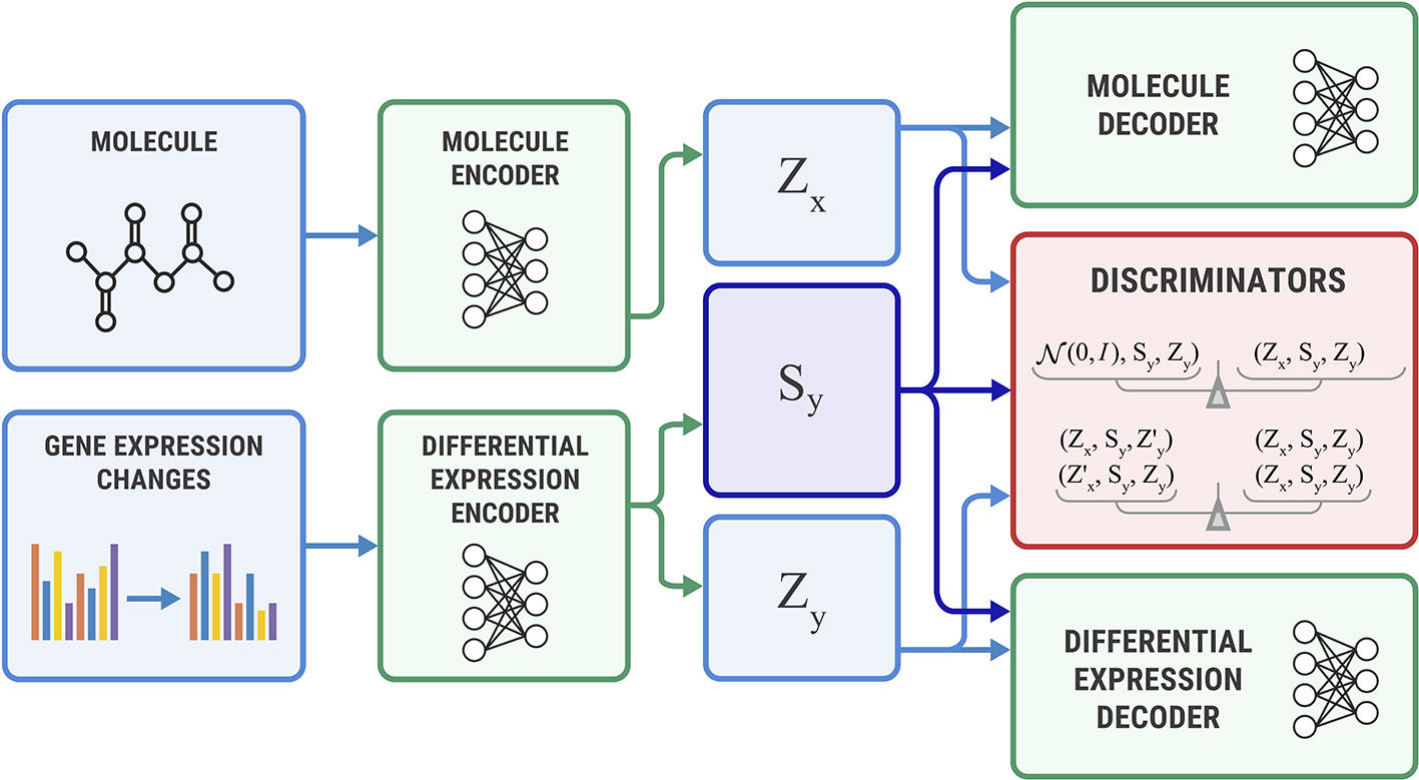
The Unidirectional Adversarial Autoencoder: a simplified version of a Bidirectional Adversarial Autoencoder for generating from ***p***(***x**|**y***). The discriminator part ensures that the three latent code components are independent, and the object’s exclusive latent code is indistinguishable from the prior distribution.

The Bidirectional AAE can generate molecules that cause given transcriptome changes and transcriptome changes caused by a given molecule. However, if we only need conditional generation of molecules *p*(*x* | *y*), we simplify the model by removing the encoder of *s_x_*. The encoder *E_x_* returns only an exclusive part: *z_x_* = *E_x_*(*x*). For this model, we derived the objective from Equation 11 by setting *s_x_* equal to *s_y_* (**Figure 5**).

## Experimental Evaluation

In this section, we have described the experimental setup and presented numerical results on the toy Noisy MNIST dataset and a LINCS L1000 dataset (Duan et al., 2014) of gene expression data.

### Noisy MNIST

We start by validating our models on the Noisy MNIST (Wang et al., 2015) dataset of image pairs (*x*, *y*), for which we know the correct features in the shared representation *s*. The image *x* is a handwritten digit randomly rotated by an angle in [−π/4,π/4]. The image *y* is also a randomly rotated version of another image containing the same digit as *x* but with strong additive Gaussian noise. As a result, the only common feature between *x* and *y* is the digit. Bidirectional and Unidirectional AAEs should learn to store only the information about the digit in *s*.

The train-validation-test splits contain 50,000, 10,000, and 10,000 samples respectively. We set the batch size to 128 and the learning rate to 0.0003, and we used the Adam (Kingma and Ba, 2015) optimizer with *β*_1_ = 0.5, *β*_2_ = 0.9 for models with adversarial training and *β*_1_ = 0.99 and *β*_1_ = 0.999 for others with a single update of autoencoders per a single update of the discriminator. Encoder and decoder architectures were the same for all models, with 12-dimensional *z_x_*, *z_y_* and 4-dimensional *s*. The encoder had 2 convolutional layers with a number of channels 1 → 32 → 16 with 2D dropout rate 0.2 followed by three fully-connected layers of size 64 → 128 → 128 → 16 with batch normalization. The decoder consisted of 2 fully connected layers followed by 3 transposed convolution layers; the discriminators have two hidden layers with 1024 → 512 units. We set the weights for ℒ_rec_ to 10 and 0.1 for ℒ_shared_. Other λ were set to 1. For Unidirectional AAE, we increased weight for ℒ_info_ to 100. For baseline models we used similar architectures. Please refer to the **Supplementary Material** for additional hyperparameters.

**Figure 6:**
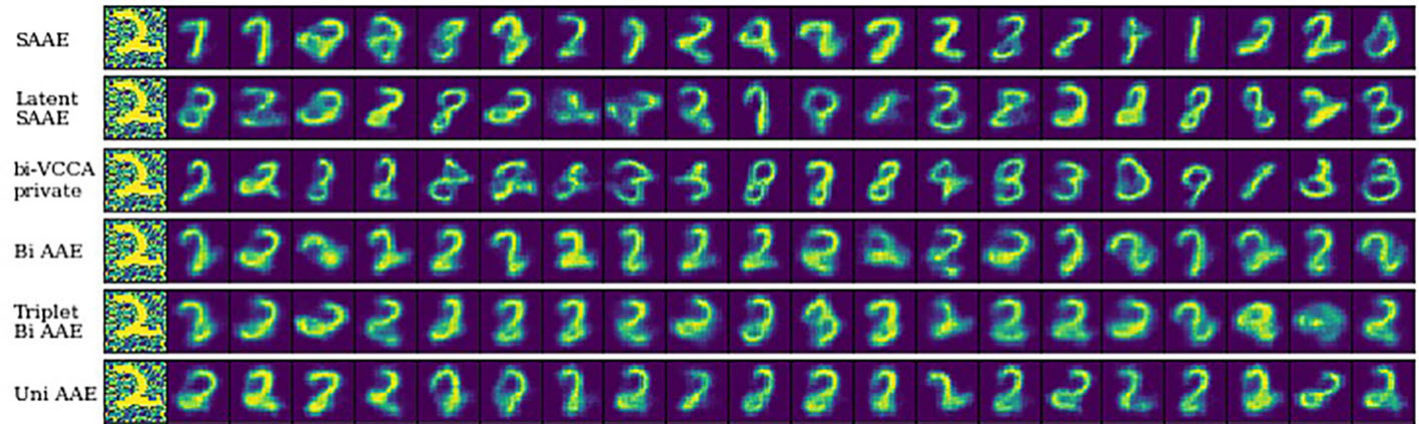
Qualitative results on a Noisy MNIST dataset. The figure shows generated images *x* for a noisy image *y* (left column) as a condition. Generated images must have the same digit as *y*.

Conditional generative model *p*(***x*** | ***y***) should produce images with the same digit as image ***y***, which we evaluate by training a separate convolutional neural network to predict the digit from *x* and comparing the most probable digit to the actual digit of *y* known from the dataset. We also estimated a conditional mutual information *ℳℐ*(***x***,***s***_***y***_|***y***) using a Mutual Information Neural Estimation (MINE) (Belghazi et al., 2018) algorithm for BiAAE, UniAAE, JMVAE, and VCCA models. For SAAE, LatentSAAE, CVAE, and VIB we estimated *ℳℐ*(***x***,***s***|***y***) since these models do not separate embeddings into shared and exclusive parts explicitly. Models with high mutual information extract relevant information from *y*. A neural network for MINE consisted of a convolutional encoder for *x* and fully-connected encoder for *s_y_*. We then passed a concatenated embedding through a fully-connected neural network to get a final estimate of mutual information. Results in **Table 1** suggest that the BiAAE model extracted relevant mutual information which, besides all, contained information about the digit of *y*. In **Figure 6**, we show example samples from the model.

**Table 1:** Quantitative results for a Noisy MNIST experiment. Conditional Generation section evaluates how often the model produced a correct digit. Latent Codes section estimates the Mutual Information between *z*_*x*_ and *s* (*y* for SAAE).

Model	Accuracy, %	MI(*x*,*s_y_*|*y*)	MI(*x*,*s*|*y*)
SAAE (Makhzani et al., 2015)	43.68	—	1.665
Latent SAAE	34.76	—	**1.681**
CVAE (Sohn et al., 2015b)	0.4583	—	0.3074
JMVAE (Suzuki et al., 2017)	5.38	0.9515	—
VIB (Alemi et al., 2017)	43.6	—	1.121
VCCA (Wang et al., 2016b)	23.35	1.239	—
BiAAE (our)	**49.21**	1.432	—
UniAAE (our)	47.61	**1.627**	—

### Differential Gene Expression

In this section, we have validated Bidirectional AAE on a gene expression profiles dataset with 978 genes. We use a dataset of transcriptomes from the Library of Integrated Network-based Cellular Signatures (LINCS) L1000 project (Duan et al., 2014). The database contains measurements of gene expressions before and after cells react with a molecule at a given concentration.

For each cell line, the training set contains experiments characterized by the control (ge_*b*_∈*ℝ*^978^) and perturbation-induced (ge_*a*_∈*ℝ*^978^) gene expression profiles. We represented molecular structures in the SMILES format (Weininger, 1988; Weininger et al., 1989). We augmented the dataset by randomly matching control and perturbation-induced measurements from the same plate.

We preprocessed the training dataset by removing molecules with a molecular weight less than 250 and more than 550 Da. We then removed molecules that did not contain any oxygen or nitrogen atoms or contained atoms besides C, N, S, O, F, Cl, Br, and H. Finally, we removed molecules that contained rings with more than eight atoms or tetracyclines. The resulting dataset contained 5,216 unique SMILES. Since the dataset is small, we pretrained an autoencoder on the MOSES (Polykovskiy et al., 2018a) dataset and used its encoder and decoder as initial weights in all models.

For all baseline models on differential gene expressions, we used similar hyperparameters shown in **Table 2** (please refer to the **Supplementary Material** for the exact hyperparameters). In all experiments, we split our dataset into train, validation, and test sets, all containing different drugs. To construct a training example, we sampled a drug-dose pair, a perturbation for this drug and dose, and a control expression from the same plate as the perturbed expression.

**Table 2:** Hyperparameters for neural networks training on gene expression data. All neural networks are fully connected, and decoders have an architecture symmetric to the encoders.

Hyperparameter	Value
Molecular Encoder	GRU; hidden size 128; 2 layers
Expression Encoder	IN(978)→256→OUT(128)
Difference Encoder	IN(129)→128→OUT(10 + 10)
Discriminator	IN→1024→512→OUT(1)
Batch Normalization	After each linear layer in encoders
Activation Function	LeakyReLU
Learning Rate	0.0003

**Figure 7:**
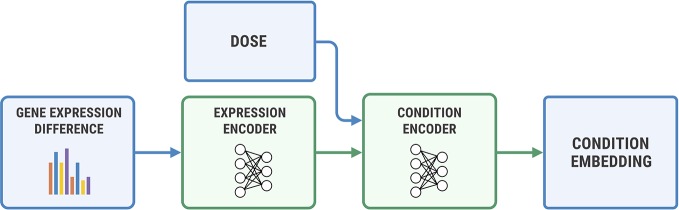
The architecture of the condition encoder for changes in the transcriptome. The input to the expression encoder is the difference between the control and perturbed expressions. We passed the dose to the last layers of the encoder.

We used a two-step encoder for ***y*** = (*η*, Δge) shown in **Figure 7**, where Δge=ge_*a*_−ge_*b*_. We first embedded Δge with a fully-connected neural network, and then concatenated the obtained representation with a logarithm of concentration *η*. We passed the resulting vector through a final encoder. The decoder has a symmetric architecture.

#### Generating Molecular Structures for Gene Expression Profiles

The proposed BiAAE model can generate molecules for given gene expression changes and vice versa. We started by experimenting with the molecular generation (**Table 3**). In the experiment, we reported a negative log-probability of generating the exact incubated drug *x* given the dose and gene expression change averaged over tokens *log* *p*(***x***|Δge,*η*). We also estimated a Mutual Information *ℳℐ*(***x***,*s*_*y*_|Δge,*η*) similar to the MNIST experiment described above. For each *η* and Δge, we generated a set of molecules *G* and estimated a fraction of valid molecules and internal diversity of *G*:

(12) $$\text{IntDiv}\left(G\right)=1-\frac{1}{\left|G\right|\left(\left|G\right|-1\right)}\sum_{\begin{array}{c} m_{1},m_{2}\in G\\ m_{1}\neq m_{2} \end{array}}T\left(m_{1},m_{2}\right),$$

**Table 3:** Validation results of conditional generation *p*(*x*|Δge,*η*).

Model	NLL	MI(*x*,*s*_*y*_|Δge,*η*)	MI(*x*,*s*|Δge,*η*)	Internal Diversity	Validity
SAAE	0.55	—	**0.11**	**0.85**	0.64
Latent SAAE	0.55	—	0.00	**0.85**	0.62
CVAE	1.22	—	0.00	0.84	0.58
JMVAE	1.42	0.00	—	0.61	**0.82**
VIB	1.46	—	0.00	0.17	0.29
VCCA	1.36	0.00	—	0.53	0.71
BiAAE	0.77	**0.32**	—	**0.85**	0.76
UniAAE	**0.53**	0.00	—	**0.85**	0.61

where *T* is a Tanimoto similarity on Morgan fingerprints. This metric shows whether a model can produce multiple candidates for a given gene expression or collapses to a single molecule.

The proposed BiAAE and UniAAE architectures show the ability to capture the dependencies in the training set and generalize to new objects from the validation set. The BiAAE model provides better mutual information while preserving valid diverse molecules.

#### Comparing Generated Molecular Structures to Known Active Molecules

In this experiment, we show that the proposed generative model (BiAAE) can produce biologically meaningful results. We used a manually curated database of bioactive molecules ChEMBL 24.1 (Gaulton et al., 2016) and additional profiles of gene expression knockdown from LINCS L1000 (Duan et al., 2014).

The first experiment evaluates molecular generation given a transcriptome change of a small molecule inhibitor of a specific protein. The ChEMBL dataset has experimental data on molecules that inhibit a certain human protein. We chose template molecules that are present in both LINCS molecule perturbation dataset and ChEMBL dataset. We used molecules that had inhibition concentration less than 10 *μ*M IC50 for only one protein.

**Figure 8:**
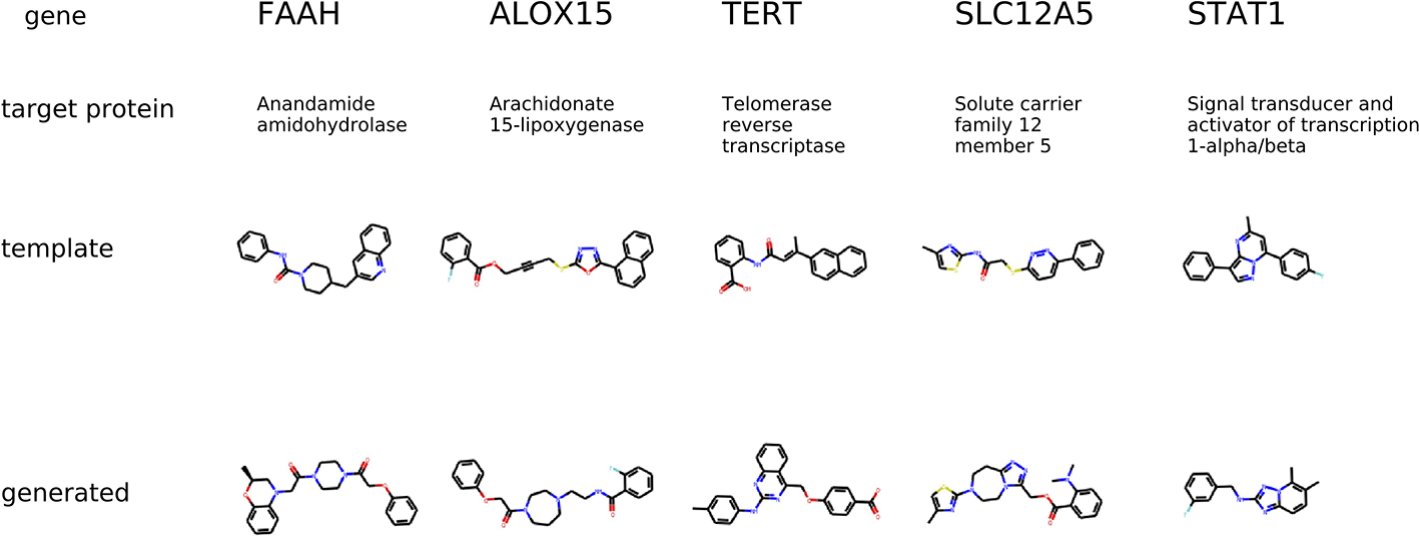
The examples of generated molecules conditioned on gene expression changes from a protein inhibitor; Real most similar inhibitors from ChEMBL are provided for comparison.

The condition for molecular generation is a transcriptome change and a dose of a template molecule. Specifically, the condition is a shared part *s_y_* of the gene expression and dose embedding. The model is expected to generate molecules that are similar to known drugs. In **Figure 8**, for several protein targets, we show a known inhibitor and generated molecules that could induce similar transcriptome profile changes.

The second experiment evaluates molecular generation given a transcriptome change of a specific gene knockdown. The LINCS dataset contains gene knockdown transcriptomes that the model was not trained on. For each gene knockdown, we found a corresponding human protein in the ChEMBL dataset. We chose template molecules that had a proven IC50 less than 10*μ*M for only one protein. The condition for molecular generation is a transcriptome change of a gene knockdown and the most common dose 10 *μ*M in LINCS. The model is expected to generate molecules that produce the same transcriptome change of gene knockdowns.

**Figure 9:**
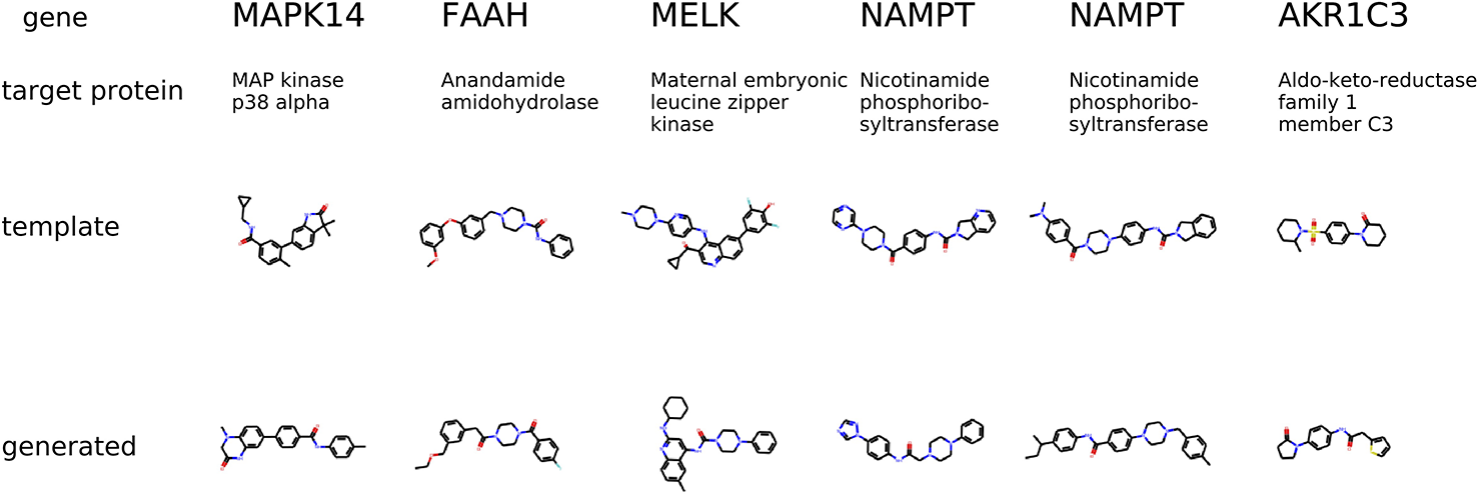
The examples of generated molecules conditioned on gene expression changes from a gene knockdown; Real most similar inhibitors of a knocked down gene are provided for comparison.

The condition is different compared to the previous experiment in a way that the gene knockdown expression profile is not induced by a small molecule but rather shows the desired behavior of the potential drug. In **Figure 9**, we show generated molecules and compare them to known inhibitors of a protein corresponding to a knocked down gene. We expect these molecules to produce similar effects in gene expression to gene knockdown.

#### Predicting Gene Expression Profiles for an Incubated Drug

We experimented with predicting gene expression changes after drug incubation (**Table 4**). First, we report estimated mutual information *ℳℐ*(Δge,*η*,***s***_***x***_|***x***) similar to the previous experiments. We also report the *R*^2^ metric, which measures the determination coefficient between the real and predicted (Δge, *η*) for a given molecule. Finally, we report a top-1 precision metric, which shows the fraction of samples for which the largest absolute change in real and predicted Δge matched.

**Table 4:** Validation results of conditional generation ***p***(***Δge***,***η***|***x***).

Model	MI(Δge,*η*,*s*_*y*_|*x*)	MI(Δge,*η*,*s*|*x*)	Top-1 precision	*R*^2^ score
SAAE	—	0.00	0.58	0.26
Latent SAAE	—	**0.23**	0.74	0.28
CVAE	—	0.01	0.29	**0.33**
JMVAE	0.00	—	0.0	0.03
VIB	—	0.00	—	—
VCCA	0.00	—	0.0	0.03
BiAAE	0.20	—	0.74	0.32
UniAAE	**0.21**	—	**0.77**	0.27

To compute *R*^2^ and top-1 precision, we only used drugs that were administered at *η* = 10 *μ*M concentration. Since we are only interested in a certain concentration, we discarded generated (Δge, *η*) tuples if *η* was far from 10 *μ*M (outside the range [−6.5,−5.5] in log_10_ scale). Note that VIB was not able to generate any gene expression changes near 10 *μ*M.

The experiment demonstrates that proposed UniAAE, BiAAE, and LatentSAAE models generalize well the symmetric task and show good metrics on predicting gene expression changes.

## Discussion

The key advantage of the proposed model compared to the previous works is the joint adversarial learning of latent representations of paired objects. This representation improves conditional generation metrics and shows promising results in molecular generation for desired transcriptome changes.

Three discriminator neural networks ensure that the latent representations divided into shared and exclusive parts are more meaningful and useful for the conditional generation. Two additional discriminator losses help the model learn a more expressive shared part and make sure that all three parts are mutually independent.

However, adversarial training slightly complicates the training procedure for the BiAAE model. In comparison with other baseline models, the training loss contains more terms, each with a coefficient to tune. In general, we tune these coefficients using grid search, and we select the best coefficients according to the generative metrics on the validation set. In practice, we simplify the grid search and use the same coefficient for the adversarial terms *λ*_1_=*λ*_4_=*λ*_5_ since the corresponding losses have values on the same scale. We choose the search space for coefficients *λ*_2_,*λ*_3_ in a way that the second and third terms provide the gradient in the same scale as the other terms.

Another problem that arises when we use the adversarial approach is the instability of training. The instability is the consequence of the minimax nature of adversarial training (Goodfellow et al., 2014). To overcome the instability, we use approaches described in (Bang and Shim, 2018), i.e., we use shallow discriminators and Adam optimizer with parameters *β*_1_=0.5,*β*_2_=0.9.

## Conclusion

In this work, we proposed a Bidirectional Adversarial Autoencoder model for the generation of molecular structures for given gene expression changes. Our AAE-based architecture extracts shared information between molecule and gene expression changes and separates it from the remaining exclusive information. We showed that our model outperforms baseline conditional generative models on the Noisy MNIST dataset and the generation of molecular structures for the desired transcriptome changes.

## Data Availability Statement

The code and datasets for this study are available at https://github.com/insilicomedicine/BiAAE.

## Author Contributions

RS and MK implemented the BiAAE and baseline models and conducted the experiments. RS, AK, and AA prepared the datasets. RS, MK, AK, and DP derived the BiAAE and UniAAE models. RS, AZ, AK, SN, and DP wrote the manuscript. AK and DP supervised the project.

## Conflict of Interest

RS, MK, AZ, AK, AA, and DP work for Insilico Medicine, a commercial artificial intelligence company. SN works for Neuromation OU, a company engaged in AI development through synthetic data and generative models.
