# Supplementary figures and images for: Molecular Generation for Desired Transcriptome Changes With Adversarial Autoencoders
Source: Front Pharmacol. 2020 Apr 17;11:269. doi: 10.3389/fphar.2020.00269 (PMC7182000; doi:10.3389/fphar.2020.00269)

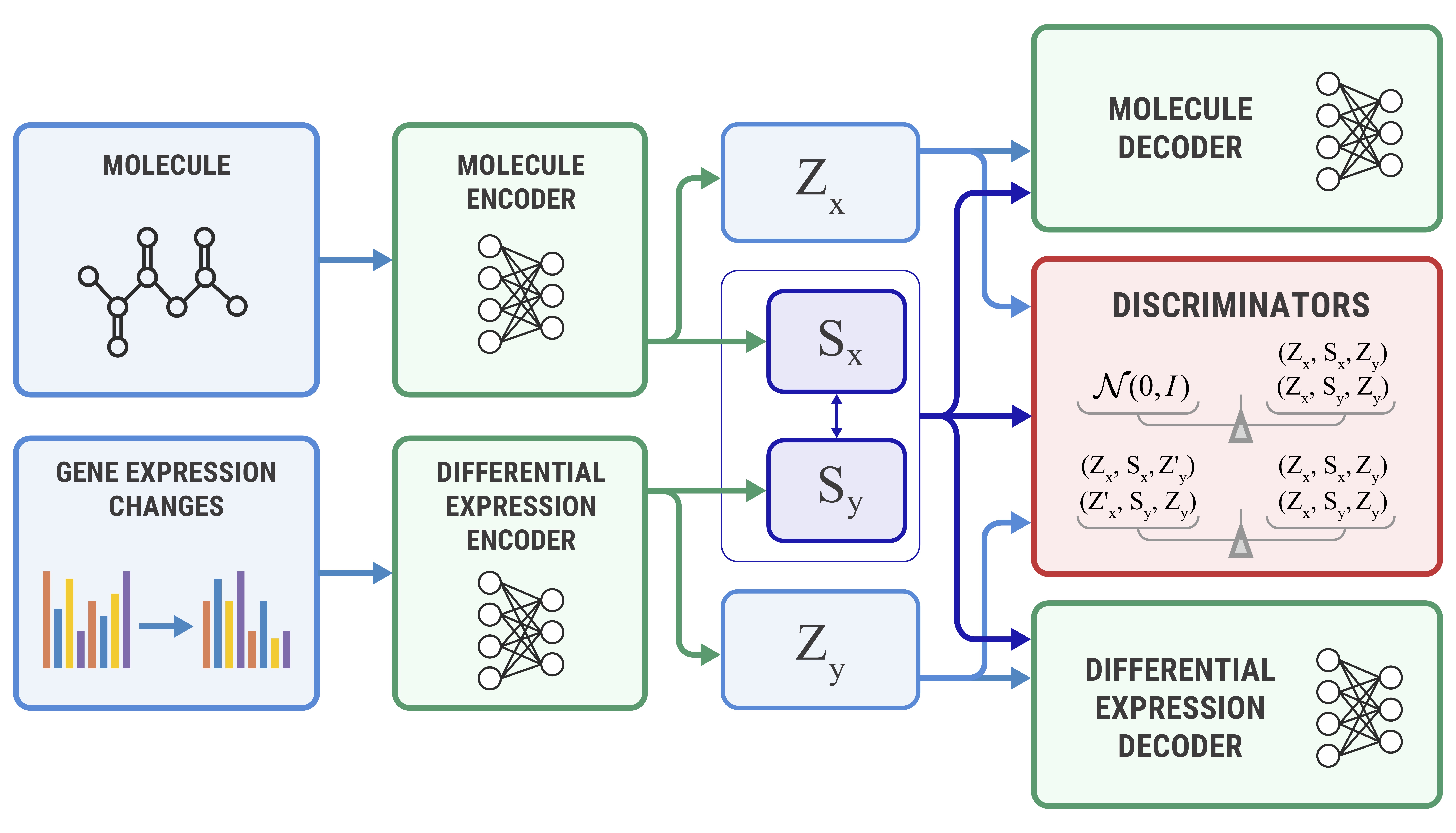

Supplement: Supplementary file 1 [file DataSheet_1.zip › biaae/images/BiAAE.jpg]
